# Coenzyme Q_0_ From *Antrodia cinnamomea* Exhibits Drug-Resistant Bacteria Eradication and Keratinocyte Inflammation Mitigation to Ameliorate Infected Atopic Dermatitis in Mouse

**DOI:** 10.3389/fphar.2019.01445

**Published:** 2019-12-03

**Authors:** Wei-Ling Chou, Tzong-Huei Lee, Tse-Hung Huang, Pei-Wen Wang, Ya-Ping Chen, Chin-Chang Chen, Zi-Yu Chang, Jia-You Fang, Shih-Chun Yang

**Affiliations:** ^1^Department of Traditional Chinese Medicine, Chang Gung Memorial Hospital, Keelung, Taiwan; ^2^Institute of Fisheries Science, National Taiwan University, Taipei, Taiwan; ^3^School of Traditional Chinese Medicine, Chang Gung University, Taoyuan, Taiwan; ^4^Graduate Institute of Health Industry Technology, Chang Gung University of Science and Technology, Taoyuan, Taiwan; ^5^School of Nursing, National Taipei University of Nursing and Health Sciences, Taipei, Taiwan; ^6^Department of Medical Research, China Medical University Hospital, China Medical University, Taichung, Taiwan; ^7^Pharmaceutics Laboratory, Graduate Institute of Natural Products, Chang Gung University, Taoyuan, Taiwan; ^8^School of Medicine, Institute of Traditional Medicine, National Yang-Ming University, Taipei, Taiwan; ^9^Chinese Herbal Medicine Research Team, Healthy Aging Research Center, Chang Gung University, Taoyuan, Taiwan; ^10^Research Center for Food and Cosmetic Safety and Research Center for Chinese Herbal Medicine, Chang Gung University of Science and Technology, Taoyuan, Taiwan; ^11^Department of Anesthesiology, Chang Gung Memorial Hospital, Taoyuan, Taiwan; ^12^Department of Cosmetic Science, Providence University, Taichung, Taiwan

**Keywords:** *Antrodia cinnamomea*, coenzyme Q_0_, atopic dermatitis, methicillin-resistant *S. aureus*, skin delivery

## Abstract

Atopic dermatitis (AD) is an inflammatory skin disease that is usually accompanied by *Staphylococcus aureus* infection due to cutaneous barrier-function damage. Benzenoid compounds from *Antrodia cinnamomea* are known to exhibit antibacterial and anti-inflammatory activities. This study sought to investigate the potential of benzenoids for treating bacteria-infected AD. The compounds were screened against methicillin-resistant *S. aureus* (MRSA). Coenzyme Q_0_ (CoQ_0_), a key ingredient in *A. cinnamomea*, showed the strongest MRSA growth inhibition. We further tested the inhibitory effect of CoQ_0_ on planktonic and biofilm MRSA. The work was also performed to explore the potential effectiveness of CoQ_0_ on AD using activated keratinocytes and *in vivo* experimental AD mice as the models. The minimum inhibitory concentration (MIC) and minimum bactericidal concentration (MBC) of CoQ_0_ against MRSA were 7.81 μg/ml. CoQ_0_ was found to eradicate biofilm MRSA efficiently and reduce the biofilm thickness. CoQ_0_ killed MRSA by inhibiting DNA polymerase and topoisomerases. A proteomic assay showed that CoQ_0_ also reduced the ribosomal proteins. In the anti-inflammation study, CoQ_0_ was found to downregulate the expression of interleukin (IL)-6, chemokine (C-C motif) ligand (CCL)5, and CCL17 in HaCaT cells. CoQ_0_ at 0.5 μg/ml could recover the filaggrin decreased by HaCaT activation to the normal control. We established a bacteria-infected AD-like model in mice using ovalbumin (OVA) and topically applied MRSA. Topical CoQ_0_ delivery lessened the MRSA presence in the AD-like lesions by >90%. The erythema, barrier function, and epidermal thickness of the AD-like wounds were improved by CoQ_0_ through the reduction of IL-1β, IL-4, IL-6, IL-10, interferon (IFN)-γ, and by neutrophil infiltration in the lesional skin. CoQ_0_ is therefore regarded as effective in mitigating AD symptoms associated with bacterial load.

## Introduction

Atopic dermatitis (AD) is a commonly diagnosed skin condition characterized by a heterogeneous pathogenesis such as chronic inflammation, barrier dysfunction, and pruritus (Roesner et al., 2016). AD generally begins in early childhood and affects 10% to 25% of children and 2% to 10% of adults (Nygaard et al., 2017). The barrier deficiency caused by AD has been linked to increased bacterial infection in the skin (Sohn, 2018). More than 90% of AD patients are colonized with *Staphylococcus aureus* (Ong, 2014). The emergence of methicillin-resistant *S. aureus* (MRSA) has led to an increase in AD exacerbation due to its resistance to current antibiotics (Shi et al., 2018). It is reported that >700,000 people die each year because of infection by resistant microbes (Franci et al., 2018). The increasing resistance of bacteria in AD and the deteriorated inflammation advocate the demand for novel anti-MRSA and anti-inflammatory agents for AD treatment.

Some investigations have acknowledged that natural products are rich sources of antibacterial and anti-inflammatory potencies. *Antrodia cinnamomea* is a fungus inhabiting the inner cavity of *Cinnamomum kanehirae* Hayata. It is a traditional medicine for treating hypertension, cirrhosis, hepatoma, diarrhea, and itchy skin (Geethangili and Tzeng, 2011). The main active ingredients in *A. cinnamomea* include terpenoids, lignans, polysaccharides, and benzenoids. The extracts and compounds of *A. cinnamomea* demonstrate the capability to inhibit skin inflammation (Amin et al., 2015; Tsai et al., 2015; Kuo et al., 2016). The constituents from *A. cinnamomea* are reported to show antimicrobial activity against both Gram-positive and Gram-negative species (Geethangili et al., 2010; Chiang et al., 2013; Lien et al., 2014). We previously isolated a series of benzenoids from *A. cinnamomea* and found the anti-inflammatory activity in stimulated macrophages *via* inducible nitric oxide synthase (iNOS) inhibition (Yang et al., 2009; Yu et al., 2016). Some scientists have also shown the usefulness of benzenoids from *A. cinnamomea* in suppressing inflammatory response (Chen et al., 2007; Buccini et al., 2014; Yen et al., 2018). Since AD is associated with inflammation, barrier deficiency, and bacterial infection, combined therapy with anti-inflammatory and antibacterial agents can be beneficial to alleviating the symptoms. Since *A. cinnamomea* effectively inhibits inflammation and bacterial growth, it is an ideal candidate for the development of anti-AD agents. We aimed to isolate benzenoid derivatives from *A. cinnamomea* to evaluate the capability to ameliorate AD through the reduction of inflammation and the MRSA burden. Our results exhibited that among all benzenoids tested, 2,3-dimethoxy-5-methyl-1,4-benzoquinone (coenzyme Q_0_, CoQ_0_) was the most effective antibacterial compound. Using human keratinocytes as the cell model, we demonstrated that CoQ_0_ could reduce the up-regulation of cytokines and chemokines. CoQ_0_ could also enhance the decrease in TJ-related proteins caused by keratinocyte stimulation. Here, we showed that topical CoQ_0_ administration remarkably improved AD symptoms and the associated MRSA burden in the mouse model.

## Materials and Methods

### Compounds

**Figure 1:**
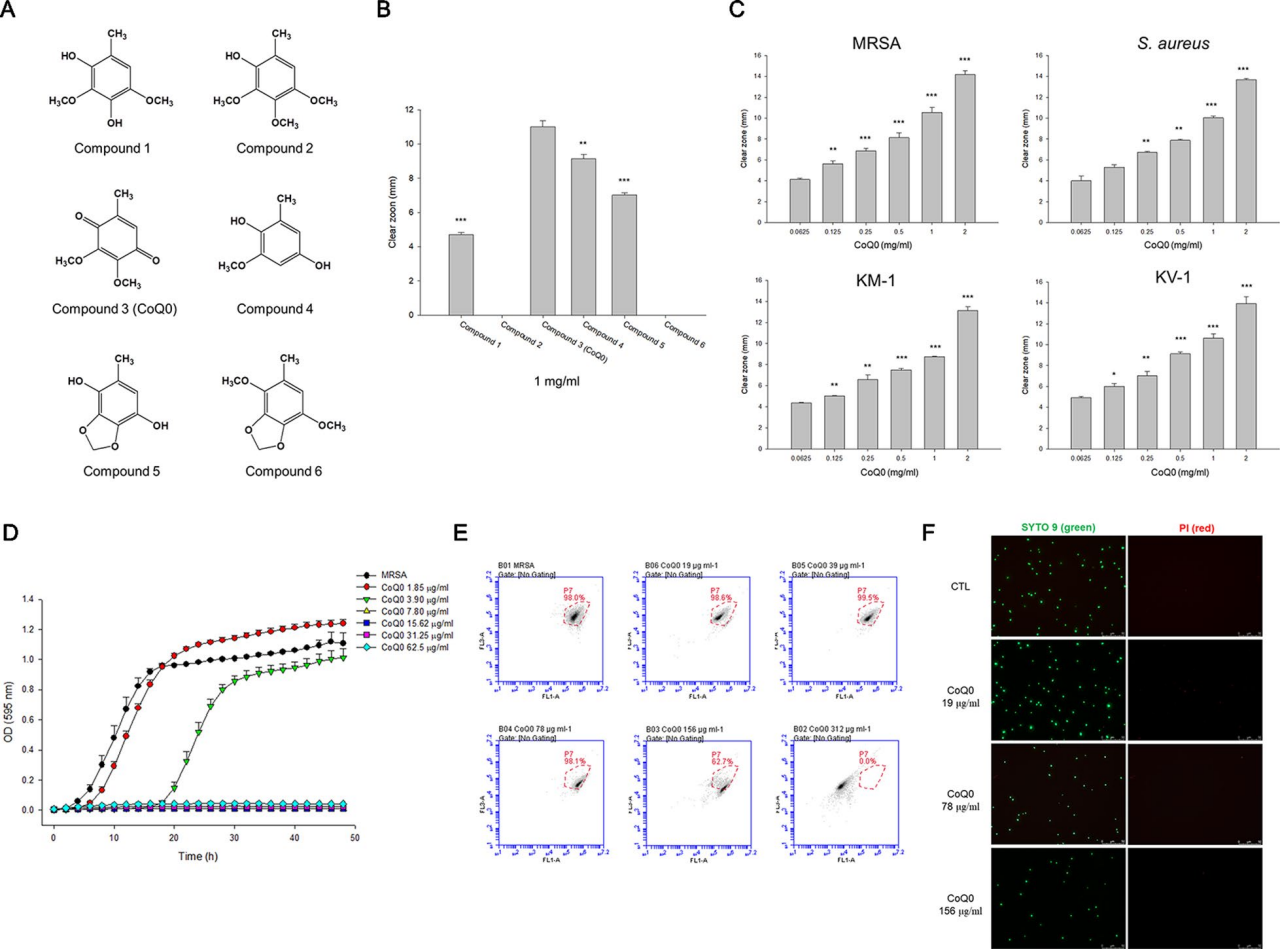
Determination of the antibacterial activity of *A. cinnamomea* extracted benzenoids. **(A)** The chemical structures of the benzenoids. **(B)** Zone of inhibition of MRSA treated by benzenoids measured from agar diffusion assay. **(C)** Zone of inhibition of MRSA, *S. aureus*, and VISA treated by CoQ_0_ measured from agar diffusion assay. **(D)** The growth curves of MRSA treated by CoQ_0_ at different concentrations within 48 h. **(E)** The planktonic live/dead MRSA strain determined by flow cytometry. **(F)** The planktonic live/dead MRSA strain viewed under fluorescence microscopy. Each value represents the mean ± SEM (n *=* 4). ***p < 0.001; **p < 0.01; *p < 0.05.

The agar-cultured mycelium of *A. cinnamomea* was used to prepare the extract in 95% ethanol for 3 days at room temperature. The mycelium of *A. cinnamomea* in an agar plate is shown in **Supplementary Figure 1**. A voucher specimen of *A. cinnamomea* has been deposited at the herbarium of the Institute of Fisheries Science, National Taiwan University. The fermentation of *A. cinnamomea* and the extraction and isolation of benzenoids were the same as in the previous study (Yang et al., 2009). All compounds were identified by nuclear magnetic resonance (NMR) and mass spectrometry. As shown in **Figure 1A**, the following six benzenoid analogs were obtained and identified: 2,4-dimethoxy-6-methylbenzene-1,3-diol (compound 1), 6-methyl-2,3,4-trimethoxyphenol (compound 2), CoQ_0_ (compound 3), 2-methoxy-6-methylbenzene-1,4-diol (compound 4), 2,3-(methylenedioxy)-6-methylbenzene-1,4-diol (compound 5), and 1,4-dimethoxy-2,3-methylenedioxy-5-methylbenzene (compound 6). The spectra of ^1^H NMR for all compounds are shown in **Supplementary Figure 2**. The purity of all compounds was over 98%. The profiles of spectrometry of each compound are listed below.

Compound 1: colorless crystals; UV (MeOH) λ_max_ (log ε) 285 (3.39) nm; IR (ZnSe) ν_max_ 3438, 2941, 1503, 1477, 1379, 1320, 1198, 1123, 1072, 898 cm^−1^; ^1^H NMR (CD_3_OD, 300 MHz): δ_H_ 2.11 (3H, s), 3.75 (3H, s), 3.80 (3H, s), 6.44 (1H, s); electron ionization mass spectra (EIMS) *m/z*: 184 [M]^+^, 105 (100), 77 (85), 71 (70).

Compound 2: amorphous powder; UV (MeOH) λ_max_ (log ε) 287 (3.57) nm; IR (ZnSe) ν_max_ 3440, 2935, 2843, 1496, 1423, 1362, 1203, 1124, 1082 cm^−1^; ^1^H NMR (CD_3_OD, 300 MHz): δ_H_ 2.15 (3H, s), 3.76 (3H, s), 3.80 (3H, s), 3.83 (3H, s), 6.50 (1H, s); EIMS *m/z*: 198 [M]^+^, 183 (25), 105 (44), 77 (64).

Compound 3: needles; UV (MeOH) λ_max_ (log ε) 265 (3.72) nm; IR (ZnSe) ν_max_ 2927, 2858, 1657, 1604, 1455, 1275, 1207, 1142, 1074, 887 cm^−1^; ^1^H NMR (CDCl_3_, 300 MHz): δ_H_ 2.01 (3H, d, *J* = 1.5 Hz), 3.97 (3H, s), 4.00 (3H, s), 6.42 (1H, q, *J* = 1.5 *Hz*); ESIMS *m/z*: 205 [M + Na]^+^.

Compound 4: colorless crystals; UV (MeOH) λ_max_ (log ε) 266 (3.82) nm; IR (ZnSe) ν_max_ 3423, 1648, 1604, 1495, 1445, 1366, 1318, 1230, 1148, 1080 cm^−1^; ^1^H NMR (CDCl_3_, 300 MHz): δ_H_ 2.18 (3H, s), 3.81 (3H, s), 6.19 (1H, d, *J* = 2.7 Hz), 6.28 (1H, d, *J* = 2.7 Hz); EIMS *m/z*: 154 [M]^+^, 98 (89), 71 (100).

Compound 5: colorless crystals; UV (MeOH) λ_max_ (log ε) 283 (3.26) nm; IR (ZnSe) ν_max_ 3250, 1641, 1475, 1398, 1365, 1283, 1214, 1124,1049, 1017 cm^−1^; ^1^H NMR (CD_3_OD, 300 MHz): δ_H_ 2.08 (3H, d, *J* = 0.7 Hz), 5.84 (2H, s), 6.16 (1H, q, *J* = 0.7 Hz); ESIMS *m/z*: 191 [M + Na]^+^.

Compound 6: colorless crystals; ^1^H NMR (CDCl_3_, 300 MHz): δ_H_ 2.16 (3H, s), 3.83 (3H, s), 3.86 (3H, s), 5.91 (2H, s), 6.28 (1H, s).

### The Strains of *S. aureus*

*S. aureus* (ATCC6538) and MRSA (ATCC33591) were purchased from American Type Culture Collection. The clinical isolates of MRSA (KM-1) and vancomycin-intermediate *S. aureus* (VISA, KV-2) were the gifts of Kaohsiung Medical University Hospital. All *S. aureus* species were grown in tryptic soy broth (TSB) at 37°C and 150 rpm.

### Agar Diffusion Assay

The agar diffusion assay was carried out by inoculating the microbes at OD_600_ = 0.7 in 0.75% tryptone soya broth (TSB) agar. The mixture of microbes and agar (5 ml) was dispersed into a dish for 15 min. The compounds (1 mg/ml, 10 µl) were dropped onto the agar. The compounds were chemically stable under the conditions used. The diameter of the clear zone without bacteria was measured after a 16-h incubation.

### Minimum Inhibitory Concentration and Minimum Bactericidal Concentration

Minimum inhibitory concentration (MIC) was measured using a broth 2-fold serial dilution technique. The microbes were diluted by TSB to obtain a concentration of OD_600_ = 0.01 (2 × 10^6^ colony-forming unit (CFU)/ml). The bacteria were exposed to several dilutions of the compounds with TSB and incubated at 37°C for 20 h. The absorbance of the mixture was detected by an enzyme-linked immunosorbent assay (ELISA) reader at 595 nm. A reading of <0.1 was recognized as MIC. The microbe suspension was diluted by phosphate-buffered saline (PBS) and plated on a TSB plate. The plate was incubated at 37°C for 20 h. The CFU was then counted. Minimum bactericidal concentration (MBC) was defined as the lowest compound concentration to kill ≥99.9% of the stains.

### Growth Curve of CoQ_0_-Treated MRSA

A broth dilution method was used to detect a MRSA growth curve within 48 h. CoQ_0_ at the concentration of 1.85 to 62.5 µg/ml was pipetted into TSB (200 µl) with MRSA (2 × 10^5^ CFU/ml). The real-time measurement of OD_595_ was monitored using a multimode plate reader (BioTek).

### MRSA Survival Detected by Flow Cytometry and Fluorescence Microscopy

MRSA was diluted by TSB to obtain an OD_600_ of 0.1. The bacteria pellet was achieved by centrifugation at 12,000 rpm for 3 min, and then suspended in culture medium with CoQ_0_ (19–312 µg/ml). The samples were stained using a Live/Dead BacLight^®^ kit after a 2-h incubation at 37°C and 150 rpm. The samples were assayed by flow cytometry (BD Biosciences) to determine MRSA viability. Fluorescence microscopy was also applied to visualize the live and dead MRSA.

### Assessing MRSA Biofilm

The construction of MRSA biofilm was described previously (Alalaiwe et al., 2018). After the CoQ_0_ (78–312 µg/ml) or cetylpyridinium chloride (CPC, 75 µg/ml) treatment for 24 h, the biofilm was rinsed using PBS to remove the loosely adherent planktonic MRSA and was then suspended in PBS. The recovered MRSA outside the biofilm and the MRSA inside the biofilm were placed in an agar plate for 24 h to count CFU. The CoQ_0_- or CPC-treated biofilm was stained using Live/Dead BacLight^®^. The 3D structure, thickness, and fluorescence of the biofilm were observed by confocal microscopy (Leica).

### MRSA Morphology Visualized by Electron Microscopy

The bacteria were grown and diluted to achieve an OD_600_ of 0.3. MRSA were treated by CoQ_0_ at 312 µg/ml for 4 h. The preparation of the samples for visualization under scanning electron microscopy (SEM) and transmission electron microscopy (TEM) was described in the previous reports (Alalaiwe et al., 2018; Yang et al., 2019).

### Total Amounts of Protein, RNA, and DNA in MRSA

MRSA were grown in TSB to OD_600_ = 3. CoQ_0_ at 156 µg/ml was added to the MRSA suspension at 37°C for 4 h. The centrifuged MRSA pellet was resuspended in water. The analysis of total protein, RNA, and DNA was performed using a Bio-Rad protein assay kit, a Direct-zol RNA miniprep kit, and a Presto Mini Bacteria kit according to the respective manufacturer’s instructions.

### Bactericidal Mechanisms of CoQ_0_

An inhibition assay against DNA polymerase, topoisomerase I, and gyrase was carried out to elucidate the possible mechanisms of anti-MRSA activity by CoQ_0_. DNA polymerase was analyzed using the anti-Taq polymerase chain reaction (PCR) approach. A wrapping assay was conducted to analyze topoisomerase and gyrase. The detailed procedures of these methods were described previously (Yang et al., 2019).

### Proteomic Analysis

The proteomic analysis method of MRSA with or without CoQ_0_ treatment was modified from the previous study (Lin et al., 2018). Briefly, proteins in equal amounts were collected from MRSA samples for iTRAQ labeling. The protein mixtures were digested with modified, sequencing-grade trypsin at 37°C for 16 h. The peptides were then labeled with iTRAQ reagent for 1 h, pooled, and desalted. The eluted peptides were dried by vacuum centrifugation. The labeled peptides were then resuspended with buffer and separated using a 2D LC-mass/mass system (Thermo Fisher). The detailed setup of the LC-mass/mass was described in Chu et al. (2019). The data analysis for the iTRAQ-labeled proteins was carried out using Proteome Discoverer software (Thermo Fisher). The mass/mass spectra were searched against the UniProt database using the Mascot search engine (Matrix Science). The datasets of proteomic analysis for this study can be found on the website jPOSTrepo (http://repository.jpostdb.org/). The accession number (announce ID) is JPST000643.

### Cytotoxicity of CoQ_0_-Treated Keratinocytes

HaCaT cell line (ATCC number: PCS-200-011) was cultured in DMEM supplemented with 10% fetal bovine serum (FBS) and 1% penicillin-streptomycin in 5% CO_2_ at 37°C. After CoQ_0_ incubation at 0.25 to 16 µg/ml for 24 h, (3-(4,5-dimethylthiazol-2-yl)-2,5-diphenyltetrazolium bromide (MTT) solution at 5 mg/ml was added to the plate for 4 h. The formazan precipitate was solubilized with DMSO and then read by an ELISA reader at 550 nm to determine keratinocyte viability.

### Activating Keratinocytes by Tumor Necrosis Factor-α and Interferon-γ

HaCaT were stimulated by **tumor necrosis factor** (TNF)-α and **interferon** (IFN)-γ (20 ng/ml) to activate the inflammatory condition. CoQ_0_ at 0.25 to 2 µg/ml was added to the cells simultaneously. After a 24-h incubation, the culture supernatant was harvested for cytokine and chemokine analysis. The cells were pipetted into the lysis buffer to gain the tight junction (TJ)-related proteins from the whole cell lysate for a Western blotting assay.

### ELISA Assay

The cytokines and chemokines released from keratinocytes were detected by the ELISA method. The levels of interleukin (IL)-6, chemokine (C-C motif) ligand (CCL)5, and CCL17 were measured using commercial kits (ELISA MAX Deluxe Set, BioLegend) based on the manufacturer’s instructions. The detailed procedures for the ELISA assay were described in our previous study (Lin et al., 2018).

### Immunoblotting Assay

The separated proteins were transferred to the nitrocellulose membrane and probed with primary antibodies against filaggrin, involucrin, integrin β1, and glyceraldehyde-3-phosphate dehydrogenase (GAPDH) overnight at 4°C. The membrane was incubated with anti-rabbit monoclonal antibody for 1 h. The bound antibody was observed using enhanced chemiluminescence reagent. The protein amount was quantified by the ratio of the densitometric measurement of the proteins to the corresponding GAPDH.

### Animals

Eight-week-old male Balb/c mice were purchased from the National Laboratory Animal Center (Taipei, Taiwan). One-week-old male or female pigs were provided by Pigmodel Animal Technology (Miaoli, Taiwan). They were housed at an ambient temperature of 25 ± 2°C and a relative humidity of 50 ± 10% on a 12-h light/dark cycle. All animals were treated in strict accordance with the recommendations set forth in the guidelines of the Institutional Animal Care and Use Committee of Chang Gung University (CGU15-083). Food and water were given *ad libitum*. All efforts were made to attenuate suffering.

### Skin Absorption of CoQ_0_

The *in vitro* Franz cell approach was used to determine cutaneous CoQ_0_ delivery into the skin. The excised pig skin was mounted between the donor and receptor with the stratum corneum (SC) facing the donor side. The donor was filled with 0.5 ml of CoQ_0_ (1 mg/ml) in 30% ethanol/pH 7.4 buffer. The receptor medium was the same as the vehicle in the donor. The penetration area available was 0.79 cm^2^. The receptor temperature was maintained at 37°C with a stirring rate of 600 rpm triggered by a stir bar. A 300-µl aliquot in the receptor was withdrawn at the determined intervals. At 24 h post-treatment, the skin was removed to evaluate the skin deposition of the compound. The skin was extracted by methanol in a MagNA Lyser homogenizer (Roche); then the homogenate was centrifuged at 10,000*g* for 10 min. All samples were analyzed to obtain the absorption level by high-performance liquid chromatography (HPLC). The stationary phase of HPLC was a 25-cm-long, 4-mm inner diameter reverse phase C18 column (Merck). The mobile phase consisted of methanol and double-distilled water (50:50) at a flow rate of 1 ml/min. The wavelength of the UV/visible detector was 240 nm.

### Establishing a Mouse Model With Combined AD-Like Lesions and MRSA Infection

This method was adapted from the previous study that created an animal model to simulate MRSA-infected AD-like skin (Yang et al., 2018). Briefly, the mouse was treated with an intraperitoneal injection of ovalbumin (OVA, 5.3 µg/kg) for 13 d and then topical OVA administration (5.3 µg/kg) for 3 d. MRSA with a volume of 100-µl (OD_600_ = 1) was dropped on a gauze for topical application on the dorsal skin on day 15. On days 16 and 17, 1 mg/ml CoQ_0_ with a volume of 100 µl in 15% ethanol/PBS was applied to the lesional skin. The dose of 1 mg/ml was selected for CoQ_0_, which was the same as the skin absorption test. CoQ_0_ showed facile penetration in this applied concentration. The mice were divided into different groups with 6 animals per group. The gross observation of the skin surface with or without CoQ_0_ intervention was accomplished using a handheld digital magnifier (M&T Optics). The level of transepidermal water loss (TEWL) was monitored by Tewameter^®^ (Courage and Khazaka). The lesional skin was excised to measure MRSA CFU and cytokines. The skin samples were sliced to a thickness of 5 µm for hematoxylin and eosin (H&E) and immunohistochemical (IHC) staining. The skin slices of formalin-fixed, paraffin-embedded sections were prepared for IHC. The sections were incubated with anti-lymphocyte antigen 6 complex locus G6D (Ly6G) or anti-filaggrin antibody for 1 h at room temperature, washed with saline containing 0.5% Tween 20, and then incubated at ambient temperature with biotinylated donkey anti-goat immunoglobulin G (IgG) for 20 min. Color reaction was observed by using the Vectastatin Elite avidin-biotin complex kit (Vector Laboratories). All photomicrographs of histopathology were taken by Leica DMi8 microscopy.

### Skin Tolerance of CoQ_0_

The safety of topically applied CoQ_0_ was assessed in the skin of healthy mice. CoQ_0_ (1 mg/ml) in 15% ethanol/PBS was applied daily (600 µl) on the dorsal skin for 4 days. The TEWL and skin pH were monitored every day. The erythema quantified by colorimetry (Yokogawa) was also detected. The skin was excised for H&E staining after a 4-day treatment.

### Statistical Analysis

The statistical difference in the data of the different treatments with or without CoQ_0_ was analyzed using the Kruskal-Wallis test. The *post hoc* test for checking individual differences was Dunn’s test. The 0.05, 0.01, and 0.001 levels of probability were taken as statistically significant.

## Results

### CoQ_0_ Shows the Strongest Activity Against MRSA Among the Benzenoids

We began the antibacterial investigation by administering the benzenoids (1 mg/ml) to MRSA in agar to calculate the inhibition zone. The results illustrated in **Figure 1B** demonstrate that CoQ_0_ showed the greatest MRSA inhibition, followed by compounds 4, 5, and 1. Compounds 2 and 6 had no effect on forming the clear inhibition zone. **Table 1** summarizes the MIC and MBC results for all compounds against MRSA. The lowest MIC and MBC (7.81 µg/ml) were exhibited in the CoQ_0_ group, followed by compounds 4 and 5. CoQ_0_ elicited a concentration-dependent inhibition of MRSA, *S. aureus*, and clinical isolates of MRSA (KM-1) and VISA (KV-2) as shown in **Figure 1C**. The antimicrobial activity of CoQ_0_ against different microorganisms was also investigated using MIC and MBC. As shown in **Table 2**, a high antibacterial potency for CoQ_0_ was observed with MIC between 7.81 and 31.25 µg/ml.

**Table 1:** The MIC and MBC of MRSA after treatment of *Antrodia cinnamomea* compounds.

Compound	MIC (μg/ml)	MBC (μg/ml)
1	125	125
2	250	500
3 (CoQ_0_)	7.81	7.81
4	15.63	31.25
5	31.25	62.5
6	500	500

**Table 2:** The MIC and MBC of *S. aureus*, MRSA, KM1 clinical strain and KV2 clinical strain after treatment of CoQ_0_.

Strain	MIC (μg/ml)	MBC (μg/ml)
MRSA	7.81	7.81
*S. aureus*	15.63∼31.25	15.63∼31.25
KM-1	31.25	31.25
KV-2	15.63∼31.25	31.25

The growth curves realized on MRSA treated with CoQ_0_ have proved the results of the inhibition zone and MIC/MBC (**Figure 1D**). CoQ_0_ suppressed MRSA viability at all concentrations tested (1.85–62.5 µg/ml) in a dose-dependent manner. CoQ_0_-treated MRSA were further stained using a Live/Dead kit to discover the survival rate by flow cytometry. The representative diagram of the live/dead strain in **Figure 1E** demonstrates a negligible MRSA killing at CoQ_0_ concentrations of ≤78 µg/ml. The survival rate of 156 and 312 µg/ml was 63% and 0%, respectively. Visual confirmation of the effect of CoQ_0_ on MRSA eradication was obtained using fluorescence microscopy. In **Figure 1F**, the green signal stained by SYTO9 represents live MRSA, whereas the red fluorescence stained by propidium iodide (PI) indicates bacterial membrane disruption resulting in a diffuse distribution in the cytoplasm. The green dots decreased following the increase of the CoQ_0_ concentration. However, the red dots were limited, although the concentration was significantly increased.

### CoQ_0_ Inhibits Biofilm Formation

**Figure 2:**
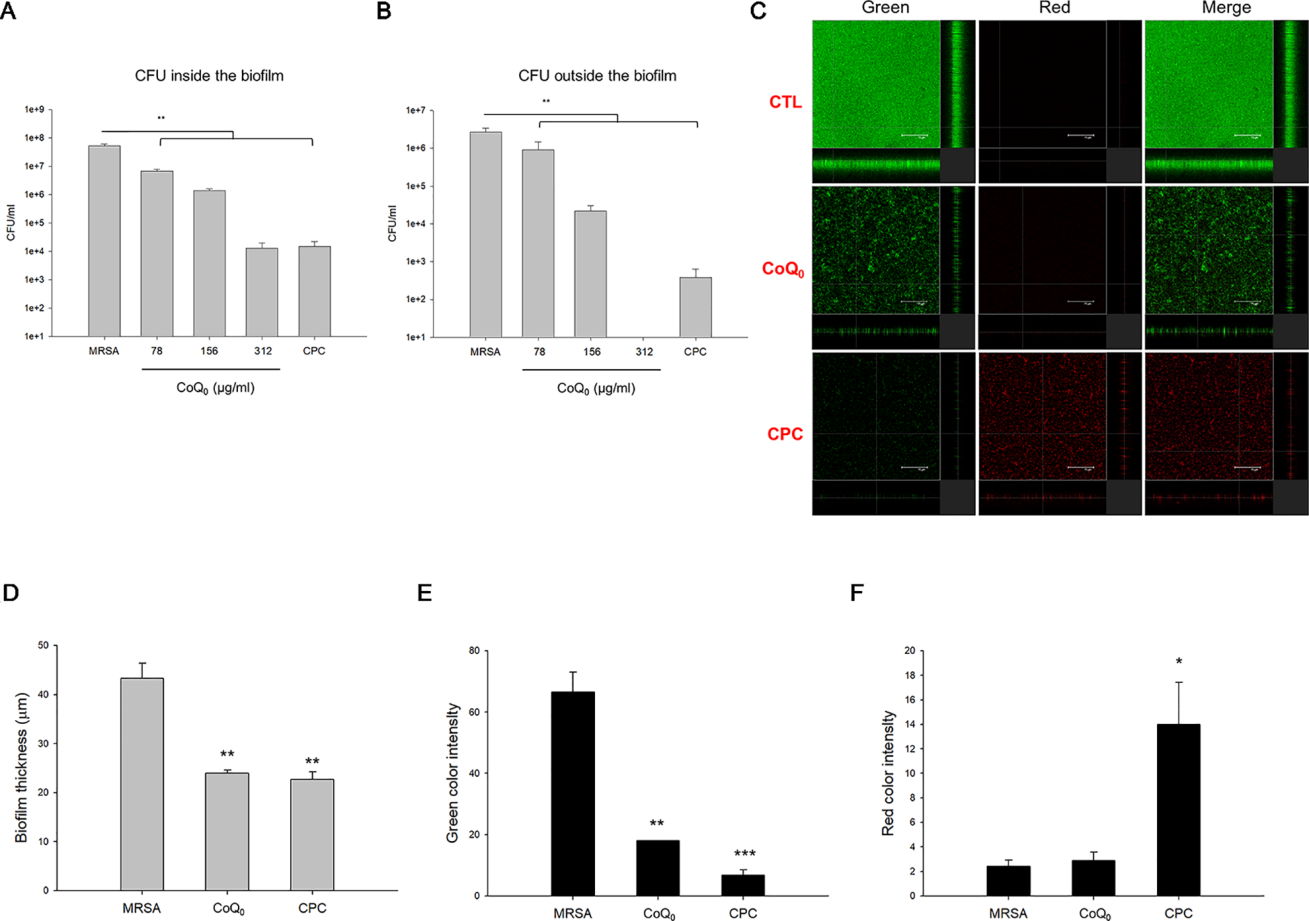
Determination of the biofilm MRSA inhibition by CoQ_0_. **(A)** MRSA CFU inside the biofilm. **(B)** MRSA CFU outside the biofilm. **(C)** The three-dimensional images of biofilm analyzed by **CLSM**. **(D)** The corresponding biofilm thickness analyzed by **CLSM**. **(E)** Quantification of green fluorescence intensity (live bacteria) of MRSA biofilm. **(F)** Quantification of red fluorescence intensity (dead bacteria) of MRSA biofilm. Each value represents the mean ± SEM (n *=* 4). ***p < 0.001; **p < 0.01; *p < 0.05.

A biofilm increases the virulence of pathogens and their resistance to antibiotics. **Figure 2A** demonstrates the corresponding number of MRSA inside the biofilm. CoQ_0_ concentration-dependently reduced the MRSA number in the biofilm. The concentration at 312 µg/ml diminished CFU by about 4 logs, leading to killing 99.99% of the MRSA population. The comparator agent CPC exhibited a reduced CFU comparable to CoQ_0_ at 312 µg/ml. The 312-µg/ml CoQ_0_ completely eradicated MRSA outside the biofilm (**Figure 2B**). This effect was greater than CPC used as the positive control. The x-y-z axis image of the biofilm showed an intact structure with condensed live MRSA inside (**Figure 2C**). The live MRSA markedly decreased when the biofilm was incubated in the presence of CoQ_0_. The CoQ_0_-treated biofilm became less uniform and looser and thinner than the intact biofilm. CoQ_0_ and CPC suppressed the biofilm’s thickness to 45% and 48% of the control, respectively (**Figure 2D**). The quantification of green intensity in the biofilm showed a significant reduction by CoQ_0_ and CPC (**Figure 2E**). CPC incubation increased the red color by about 6-fold (**Figure 2F**). However, the increase in red color was limited for CoQ_0_. This indicates that the bacterial-membrane disruption for the cytoplasm material leakage or exogenous material entrance was not the predominant mechanism for CoQ_0_.

### CoQ_0_ as the Inhibitor of DNA Polymerase and Topoisomerases for MRSA Eradication

**Figure 3:**
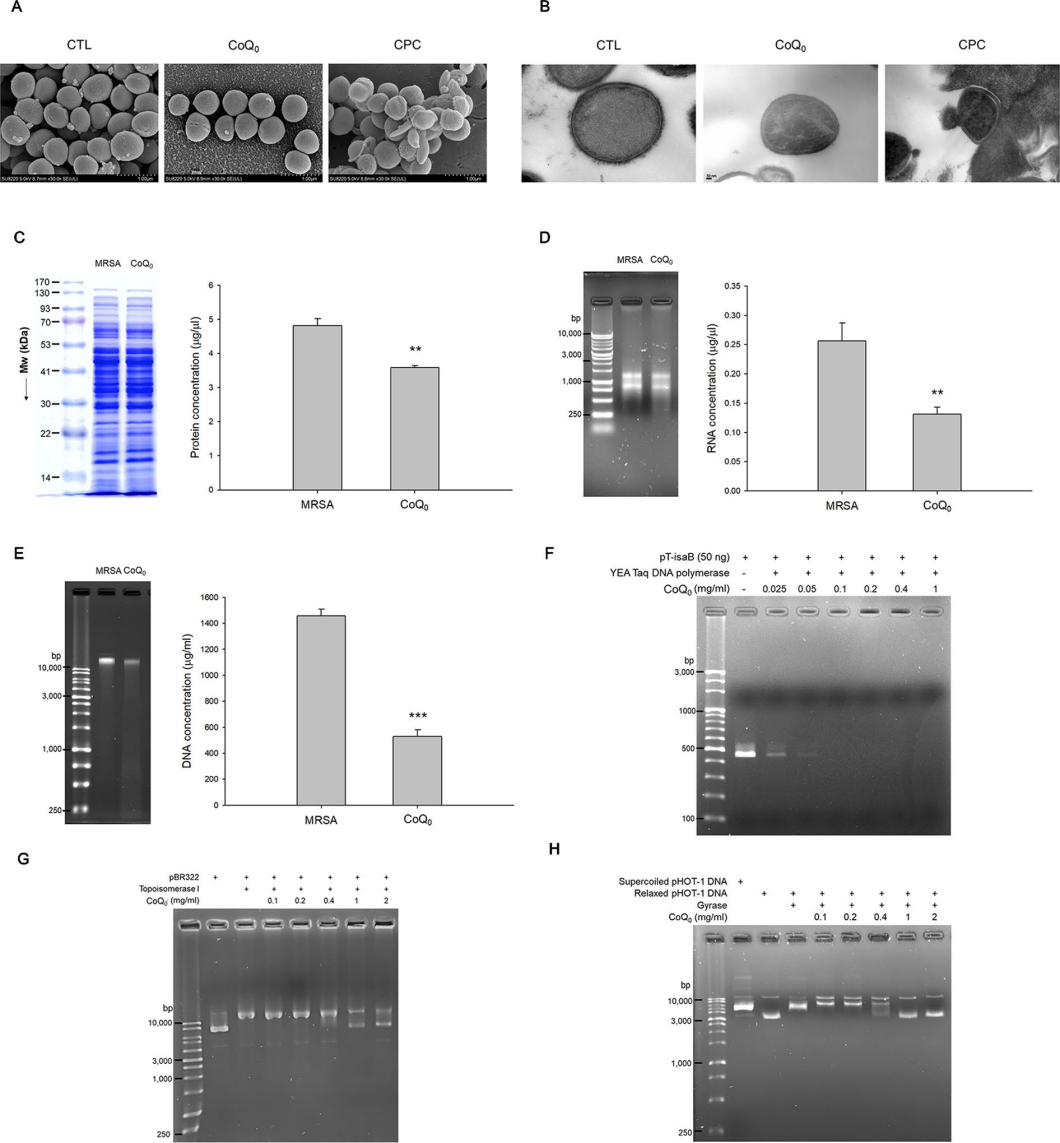
Anti-MRSA mechanisms of CoQ_0_. **(A)** Morphological changes of MRSA viewed under SEM. **(B)** Morphological changes of MRSA viewed under TEM. **(C)** Total protein amount in MRSA. **(D)** Total RNA amount in MRSA. **(E)** Total DNA amount in MRSA. **(F)** Taq DNA polymerase in PCR. **(G)** Topoisomerase I in wrapping assay; **(H)** DNA gyrase in wrapping assay. Each value represents the mean ± SEM (n *=* 4). ***p < 0.001; **p < 0.01.

The morphology of MRSA examined by SEM showed a minimal change after CoQ_0_ treatment (**Figure 3A**). Disruption of the bacterial membrane was observed by CPC, with the presence of a rough surface, irregular shape, and some cavities. As shown in the TEM image in **Figure 3B**, a large amount of cytosol was released from the porous membrane by CPC. The use of CoQ_0_ resulted in the MRSA membrane remaining intact. The total protein, total RNA, and genomic DNA of MRSA were analyzed as illustrated in **Figures 3C–E**, respectively. CoQ_0_ caused a significant decrease in the total amount of protein, RNA, and DNA compared to the untreated control. DNA polymerase is a major enzyme for DNA replication in MRSA. CoQ_0_ was evaluated for targeting enzyme inhibition using the anti-Taq PCR method to observe DNA polymerase inhibition. As shown in **Figure 3F**, the PCR products were lessened by CoQ_0_ in a concentration-dependent manner. The relaxing activity of topoisomerase I on MRSA was tested as shown in **Figure 3G**. CoQ_0_ displayed a capacity to deactivate topoisomerase I. This result was also detectable in topoisomerase II (gyrase). **Figure 3H** provides the evidence that CoQ_0_ retarded the supercoiling activity of DNA gyrase.

The qualitative and quantitative analysis of the proteomics was conducted by mass as summarized in **Table 3**. The top ten proteins with the highest or lowest concentration ratios to the control were selected to be shown in this table. The most upregulated protein by CoQ_0_ treatment was alkyl hydroperoxide reductase. Some ATP-related proteins such as ATP-dependent zinc metalloprotease, GrpE, nucleoside diphosphate kinase, and Clpb were upregulated by CoQ_0_. On the other hand, the ribosomal proteins including L13, S6, S4, L21, S18, and L31 were downregulated after CoQ_0_ treatment.

**Table 3:** Quantitative proteomics by iTRAQ labeling. Differentially expressed proteins follow the treatment of CoQ_0_.

No.	^a^Accession	Protein	MW [kDa]	Coverage	Matchedpeptides	^b^Ratios to control	Biological function
						CoQ_0_	
1.	A0A0E0VTX0	Alkyl hydroperoxide reductase AhpD	19.9	12.35	2	2.558	Antioxidant protein with alkyl hydroperoxidase activity. Required for the reduction of the AhpC active site cysteine residues and for the regeneration of the AhpC enzyme activity.
2.	A0A0E0VLN4	ATP-dependent zinc metalloprotease FtsH	77.8	3.01	2	2.541	Acts as a processive, ATP-dependent zinc metallopeptidase for both cytoplasmic and membrane proteins. Plays a role in the quality control of integral membrane proteins.
3.	A0A0E0VQR7	Delta-lysin	5.1	24.44	12	2.177	Hemolysis by symbiont of host erythrocytes.
4.	A0A0E0VPI1	Protein GrpE	24.0	31.73	7	1.749	GrpE releases ADP from DnaK; ATP binding to DnaK triggers the release of the substrate protein, thus completing the reaction cycle. Several rounds of ATP-dependent interactions between DnaJ, DnaK and GrpE are required for fully efficient folding.
5.	A0A0E0VNU7	Glutamate dehydrogenase	47.3	24.53	12	1.742	Nucleotide binding oxidoreductase activity, acting on the CH-NH2 group of donors, NAD or NADP as acceptor.
6.	A0A0E0VP69	Nucleoside diphosphate kinase	16.6	40.94	6	1.684	Major role in the synthesis of nucleoside triphosphates other than ATP. The ATP gamma phosphate is transferred to the NDP beta phosphate via a ping-pong mechanism, using a phosphorylated active-site intermediate.
7.	A0A0E0VNX4	Ribosome hibernation promoting factor	22.2	16.84	3	1.679	Required for dimerization of active 70S ribosomes into 100S ribosomes; when added to monomeric 70S ribosomes stimulates formation of 100S dimeric ribosomes. Unlike E.coli, 100S ribosomes are present during exponential growth, peak during early stationary phase and then decrease (shown for strain NBRC 3060).
8.	A0A0E0VNY8	Regulatory protein Spx	16.9	46.15	5	1.674	Interferes with activator-stimulated transcription by interaction with the RNA polymerase alpha-CTD. May function to globally reduce transcription of genes involved in growth- and development-promoting processes and to increase transcription of genes involved in thiol homeostasis, during periods of extreme stress.
9.	A0A0E0VRS6	Purine nucleoside phosphorylase DeoD-type	25.9	31.36	5	1.626	Catalysis of the reaction: purine nucleoside + phosphate = purine + alpha-D-ribose 1-phosphate. Cleavage of guanosine or inosine to respective bases and sugar-1-phosphate molecules.
10.	A0A0E0VMH0	Clpb	19.2	25.30	2	1.604	Part of a stress-induced multi-chaperone system, it is involved in the recovery of the cell from heat-induced damage, in cooperation with DnaK, DnaJ and GrpE. ATP binding
11.	A0A0E0VS50	50S ribosomal protein L13	16.3	46.21	11	0.475	This protein is one of the early assembly proteins of the 50S ribosomal subunit, although it is not seen to bind rRNA by itself. It is important during the early stages of 50S assembly.
12.	A0A0E0VL11	30S ribosomal protein S6	11.6	60.20	10	0.469	Binds together with S18 to 16S ribosomal RNA.
13.	A0A0E0VPR0	30S ribosomal protein S4	23.0	41.50	9	0.468	One of two assembly initiator proteins for the 30S subunit, it binds directly to 16S rRNA where it nucleates assembly of the body of the 30S subunit.
14.	A0A0E0VS61	Translation initiation factor IF-1	8.3	25.00	2	0.443	One of the essential components for the initiation of protein synthesis. Stabilizes the binding of IF-2 and IF-3 on the 30S subunit to which N-formylmethionyl-tRNA(fMet) subsequently binds. Helps modulate mRNA selection, yielding the 30S pre-initiation complex (PIC). Upon addition of the 50S ribosomal subunit, IF-1 is released leaving the mature 70S translation initiation complex.
15.	A0A0E0VNV5	Ribonucleoside-diphosphate reductase beta chain	38.8	14.37	3	0.424	Provides the precursors necessary for DNA synthesis. Catalyzes the biosynthesis of deoxyribonucleotides from the corresponding ribonucleotides
16.	A0A0E0VPU9	Glutathione peroxidase	18.1	15.82	2	0.409	Glutathione peroxidase is the general name of an enzyme family with peroxidase activity whose main biological role is to protect the organism from oxidative damage.
17.	A0A0E0VQU6	50S ribosomal protein L21	11.7	44.76	6	0.400	This protein binds to 23S rRNA in the presence of protein L20.
18.	A0A0E0VLB0	30S ribosomal protein S18	9.3	20.00	2	0.399	Binds as a heterodimer with protein S6 to the central domain of the 16S rRNA, where it helps stabilize the platform of the 30S subunit.
19.	A0A0E0VN53	Cold shock protein	7.4	78.79	12	0.395	Cold shock proteins are multifunctional RNA/DNA binding proteins, characterized by the presence of one or more cold shock domains.
20.	A0A0E0VQS6	50S ribosomal protein L31 type B	9.7	46.43	8	0.277	While neither of the L31 paralogs is essential, this protein does not seem to function as the main L31 protein. Has a higher affinity for 70S ribosomes than the zinc-containing L31 paralog; is able to displace it to varying extents, even under zinc-replete conditions.

a The MS/MS spectra were searched against the UniProt database (extracted for Staphylococcus aureus) using the Mascot search engine (Matrix Science, London, UK; version 2.5).

b Ratios to control indicated the fold changes in protein volume among CoQ_0_ treated samples versus MRSA samples. The top ten higher ratios mean the proteins whose expression levels were increased upon treatments of compounds, while the top ten lower ratios indicate the proteins were downregulated under the exposure to compounds.

### CoQ_0_ Is Effective in Inhibiting Inflammation and Recovering TJ Deficiency in an HaCaT Model

**Figure 4:**
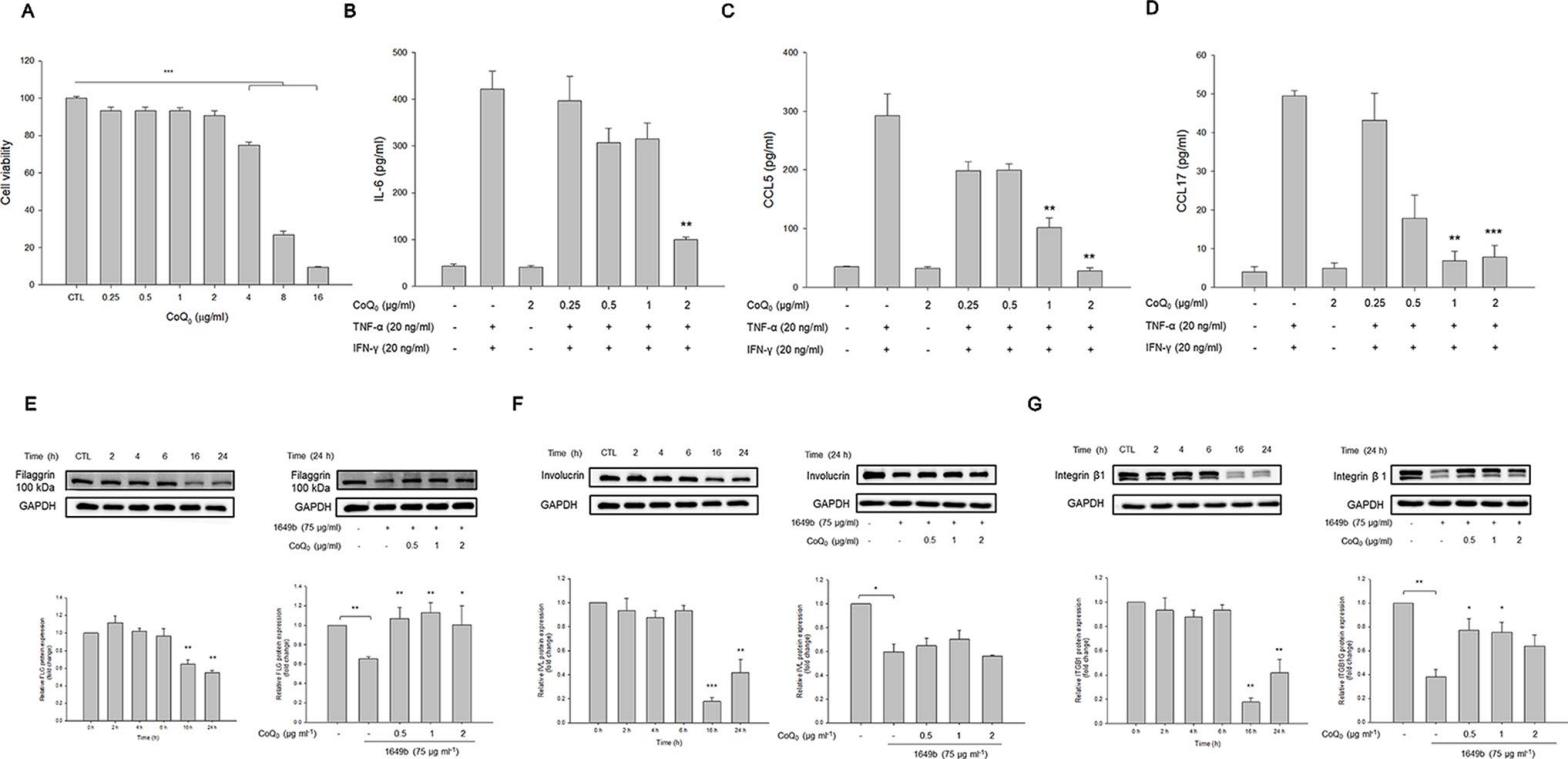
CoQ_0_ suppresses TNF-α- and IFN-γ-stimulated cytokines/chemokines and recovers TJ-protein deficiency in HaCaT cells. **(A)** The cell viability measured by MTT assay. **(B)** IL-6 in stimulated HaCaT measured by ELISA. **(C)** CCL5 in stimulated HaCaT measured by ELISA. **(D)** CCL17 in stimulated HaCaT measured by ELISA. **(E)** Filaggrin in stimulated HaCaT measured by immunoblotting. **(F)** Involucrin in stimulated HaCaT measured by immunoblotting. **(G)** Integrin β1 in stimulated HaCaT measured by immunoblotting. Each value represents the mean ± SEM (n *=* 4). ***p < 0.001; **p < 0.01; *p < 0.05.

As shown in **Figure 4A**, CoQ_0_ at the concentrations of ≤2 µg/ml did not exhibit considerable toxicity toward HaCaT. This compound decreased cell viability in a concentration-dependent fashion with significance at 4, 8, and 16 µg/ml. A noncytotoxic concentration of CoQ_0_ (0.25–2 µg/ml) was taken for further studies. Cytokines and chemokines were evaluated as biomarkers of inflammatory HaCaT. As presented in **Figure 4B**, combined TNF-α and IFN-γ greatly increased the level of IL-6 secreted by HaCaT compared to the level in the untreated control. The inhibition of IL-6 production was detected only at 2 µg/ml for CoQ_0_. The stimulators activated chemokines, including CCL5 and CCL17 (**Figures 4C, D**). CoQ_0_ dose-dependently suppressed the CCL5 and CCL17 protein levels.

Skin-barrier dysfunction and its deficiency are key features of AD. We examined the influence of CoQ_0_ on TJ-related proteins in the activated keratinocytes. Since TNF-α and IFN-γ as the stimulators showed a limited capacity to impair filaggrin, involucrin, and integrin β1 in HaCaT, the particulate matter (PM) pollutant standard (1649b) was utilized to achieve this aim according to the previous study (Lee et al., 2016). As illustrated in the left panel of **Figure 4E**, 1649b reduced filaggrin following the increase in incubation time. As shown in the right panel of **Figure 4E**, CoQ_0_ at 0.5 µg/ml was sufficient to recover filaggrin in 1649b-stimulated HaCaT to the normal baseline. The level of involucrin decreased after 1649b treatment, and CoQ_0_ could not promote involucrin production in the activated HaCaT (**Figure 4F**). CoQ_0_ improved the decreased protein level of integrin β1 by 1649b (**Figure 4G**).

### CoQ_0_ Facilely Penetrates Into the Skin

**Figure 5:**
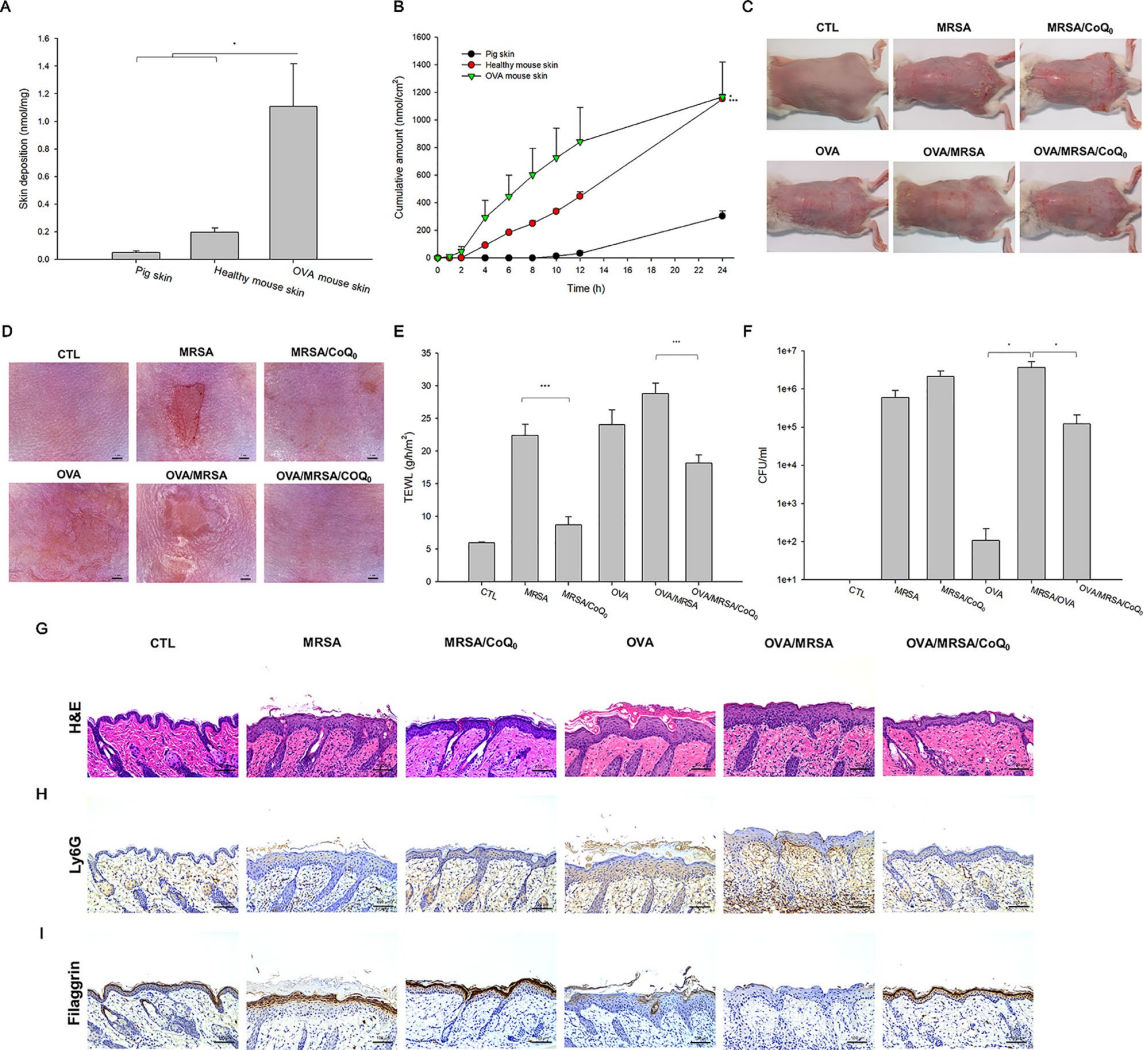
In vitro skin absorption of CoQ_0_ and *in vivo* topical application of CoQ_0_ against MRSA-infected AD-like lesion. **(A)** In vitro skin deposition of CoQ_0_ in Franz cell using pig, healthy mouse, and OVA-treated mouse skins. **(B)** In vitro skin permeation to receptor of CoQ_0_ in Franz cell using pig, healthy mouse, and OVA-treated mouse skins. **(C)** The gross images of mouse back skin. **(D)** The close-up imaging by handheld digital microscopy. **(E)** TEWL of mice skin. **(F)** MRSA CFU of mice skin. **(G)** Skin sections represented by H&E staining. **(H)** Skin sections represented by Ly6G staining for observing neutrophils. **(I)** Skin sections represented by filaggrin staining for observing TJ distribution. Each value represents the mean ± SEM (n *=* 6). ***p < 0.001; *p < 0.05.

The capability of topically applied CoQ_0_ to penetrate into the skin was carried out *in vitro* in Franz cell. As presented in **Figure 5A**, pig skin showed the minimal CoQ_0_ deposition because of its thicker and more rigid structure than rodent skin. The skin deposition indicated the uptake within the skin. CoQ_0_ uptake in healthy and AD-like mouse skin was 0.20 and 1.11 nmol/mg, respectively. This revealed a weak barrier of AD-like skin as proved by TJ deficiency. The tendency of CoQ_0_ penetration across the skin for three skin barriers was the same as that of the absorption into the skin reservoir (**Figure 5B**).

### CoQ_0_ Ameliorates MRSA-Infected AD-Like Lesions *In Vivo*

The AD-like lesions in the mouse model were developed by both intraperitoneal and topical OVA. MRSA were topically applied on the skin for cutaneous infection. **Figures 5C, D** show the gross and microscopic appearance of en face mouse skin, respectively. MRSA infection led to the observation of the open wound and scaling. These symptoms were partly attenuated in the mice dosed with CoQ_0_. The OVA challenge displayed atopic skin symptoms, including redness, excoriation, hemorrhage, and lichenification. The MRSA-infected OVA-treated skin showed the combined appearance of infection and AD-like lesions. CoQ_0_ exhibited some lesion suppression in the MRSA-infected AD-like skin. TEWL, as an indicator of the skin-barrier nature, showed a 4-fold increase in MRSA-infected skin compared to the untreated control (**Figure 5E**). As seen, CoQ_0_ recovered the TEWL of the infected skin from 22.4 to 8.7 g/h/m^2^. OVA treatment further weakened the barrier function of the infected skin. Topical CoQ_0_ administration markedly suppressed TEWL to 63% of the OVA/MRSA group. **Figure 5F** depicts the MRSA count in the mouse skin with different treatments. Bacterial infection increased the MRSA burden in the skin from 0 to 5.98 × 10^5^ CFU/ml. CoQ_0_ did not reduce the MRSA load in the infected skin. Because of the impaired barrier of the OVA-treated skin, a greater MRSA amount was found in the AD-like lesions than in the intact skin. CoQ_0_ reduced the pathogen burden in the OVA/MRSA group relative to the vehicle control.

Based on histological evaluation as shown in **Figure 5G**, the skin infected with MRSA exhibited the features of increased epidermis thickness and inflammatory infiltrate in the dermis. CoQ_0_ treatment on the infected skin significantly reduced these symptoms. OVA-induced AD exhibited hyperkeratosis, hypertrophy, and a thicker epidermis in the microscopic image. These symptoms were also visualized in the OVA/MRSA group accompanied with immune-cell infiltration and the possibility of MRSA accumulation in the dermis. Topical CoQ_0_ showed symptom amelioration of the MRSA-infected AD-like lesions. The epidermal thickness after CoQ_0_ treatment was 2-fold lower than the OVA/MRSA without CoQ_0_ intervention. **Figure 5H** reveals neutrophil infiltration in Ly6G-stained skin. This neutrophil migration could be hindered by topical CoQ_0_. As with HaCaT filaggrin evaluation, CoQ_0_ recovered filaggrin in the upper epidermis of the MRSA-infected skin with or without OVA intervention (**Figure 5I**).

**Figure 6:**
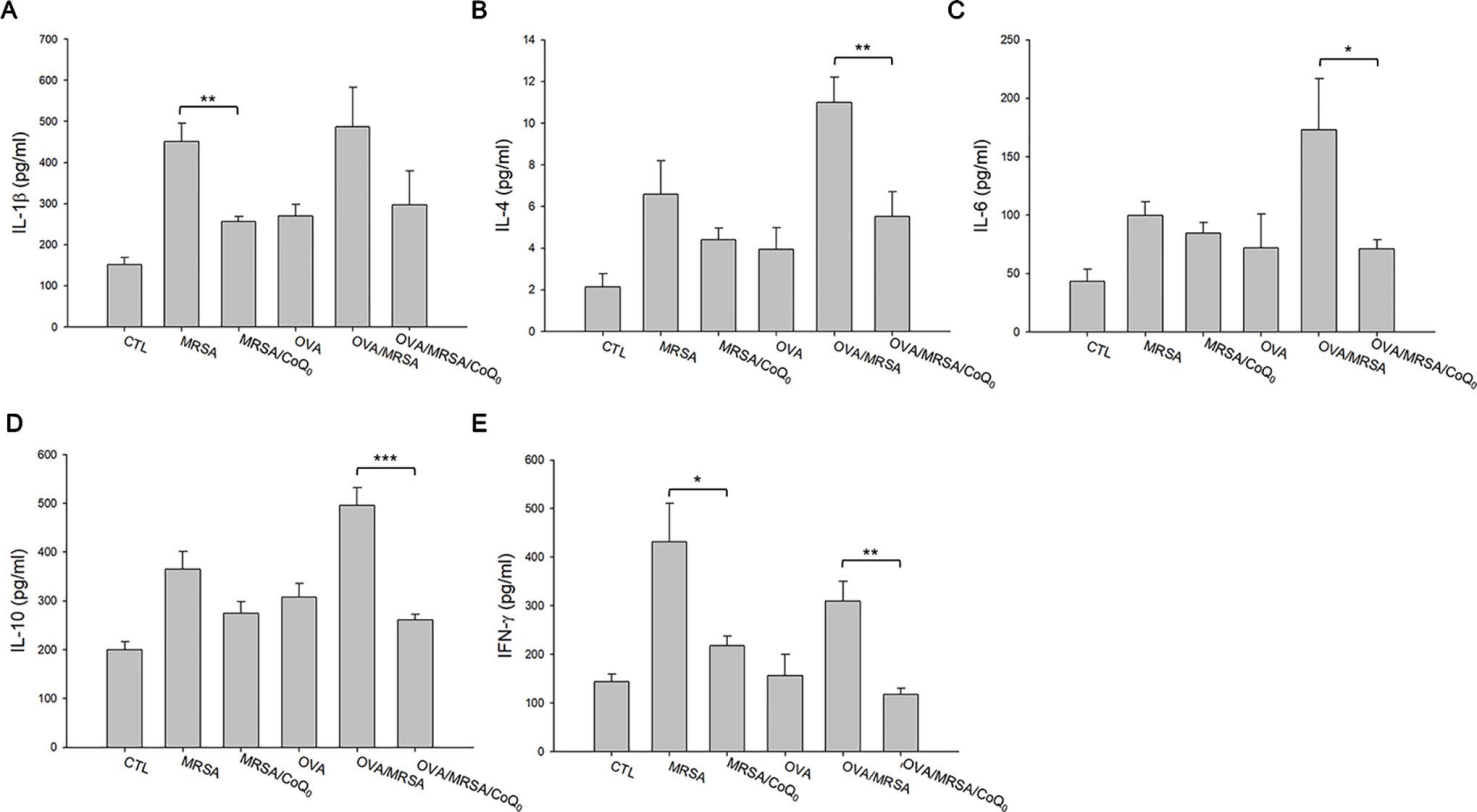
CoQ_0_ inhibits AD-like mouse skin inflammation based on cytokine assay. **(A)** IL-1β. **(B)** IL-4. **(C)** IL-6. **(D)** IL-10. **(E)** IFN-γ. Each value represents the mean ± SEM (n *=* 6). ***p < 0.001; **p < 0.01; *p < 0.05.

We next determined the presence of the cytokines in the different groups of mice as shown in **Figures 6A–E**. All cytokines were elevated by MRSA invasion, indicating the induction of cutaneous inflammation. Topical CoQ_0_ application significantly inhibited IL-1β and IFN-γ in the MRSA-infected mice. CoQ_0_-treated infected skin showed similar levels of IL-4, IL-6, and IL-10 as vehicle control after statistical assay, although the concentration with CoQ_0_ treatment was lower than that with vehicle treatment. The OVA/MRSA group generally revealed a greater increase of these cytokines than OVA or MRSA treatment alone, except the comparable IFN-γ level between the OVA/MRSA and MRSA groups. As shown in **Figures 6B–E, C**oQ_0_ significantly attenuated the IL-4, IL-6, IL-10, and IFN-γ expression levels by 50%, 59%, 47%, and 62% in mouse skin with combined OVA and MRSA, respectively.

### CoQ_0_ Elicits a Slight Skin Irritation

**Figure 7:**
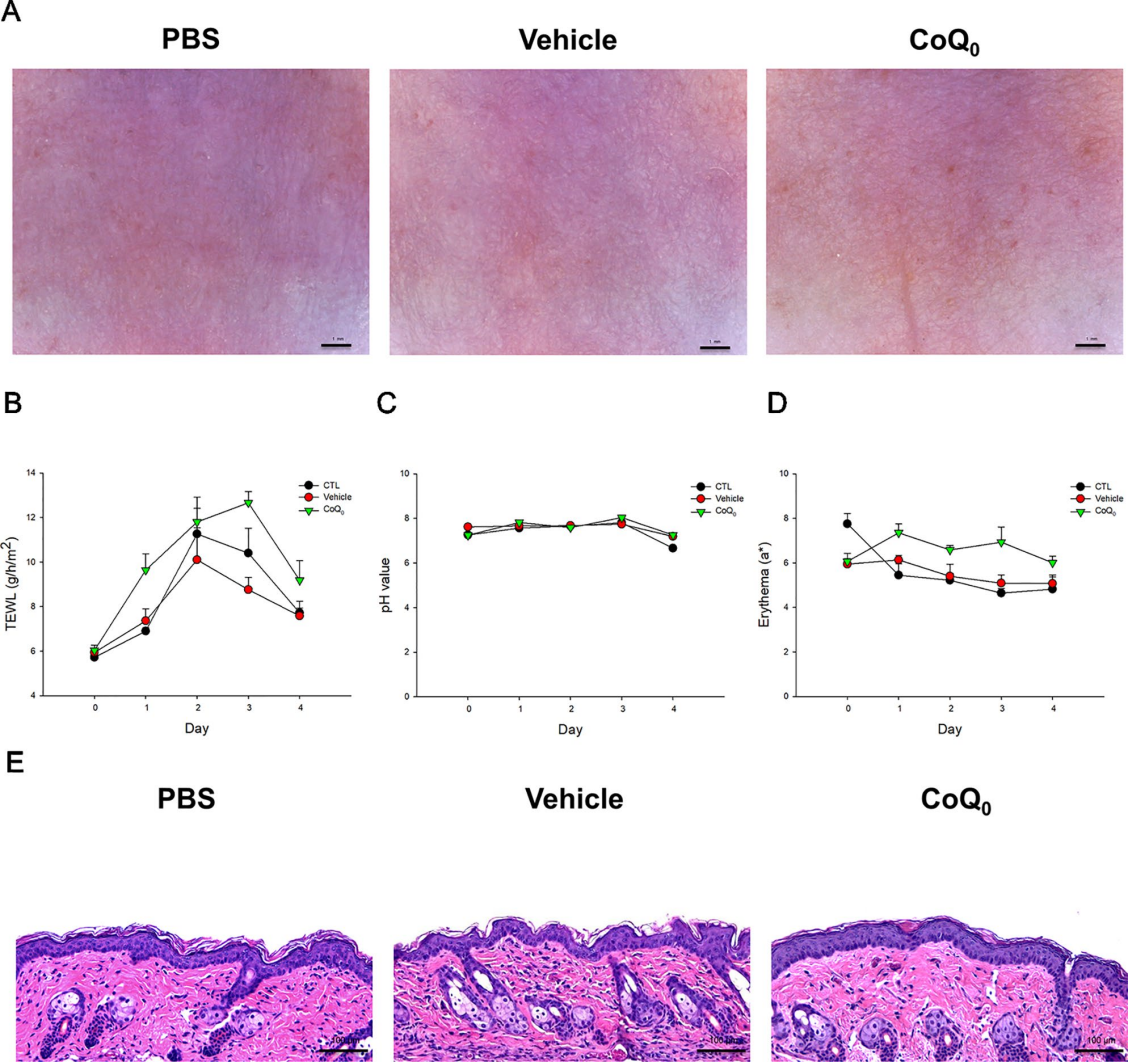
*In vivo* skin tolerance examination after a 4-day application of topically applied CoQ_0_ on mice. **(A)** The close-up imaging by handheld digital microscopy. **(B)** TEWL measurement. **(C)** Skin surface pH value. **(D)** Erythema measurement. **(E)** Skin sections represented by H&E staining. Each value represents the mean ± SEM (n *=* 6).

We attempted to determine whether topical CoQ_0_ induced adverse effects on mouse skin. As depicted in **Figure 7A**, no visible erythema or erosion occurred when the skin was applied with CoQ_0_ as compared to the PBS control and vehicle only (15% ethanol/PBS). A significant elevation of TEWL was found after PBS and vehicle treatments at Day 2 because of the skin hydration by water molecules (**Figure 7B**). This elevation decreased after a 2-day administration. CoQ_0_ application slightly increased the TEWL value as compared to vehicle control. This increase could be classified as mild. There was no difference in the skin pH and erythema quantification between the CoQ_0_ and vehicle groups (**Figures 7C, D**). The H&E-stained histology also indicated that CoQ_0_ did not irritate the skin (**Figure 7E**).

## Discussion

AD lesions can be infected by *S. aureus*. Management of the invasion of *S. aureus*, particularly MRSA, in AD is a major challenge in clinical conditions. Novel antibacterial and anti-inflammatory molecules are urgently needed to efficiently treat AD and the associated MRSA attack. We found that most of the benzenoid derivatives extracted from *A. cinnamomea* showed anti-MRSA activity. The anti-MRSA effect was quite different among different benzenoids. The greatest MRSA inhibition by CoQ_0_ implied that the presence of the benzoquinone structure was of utmost importance for anti-MRSA activity. We calculated the physicochemical properties of the compounds by molecular modeling as shown in the **Supplementary Table 1**. CoQ_0_ was the compound with the least lipophilicity according to Alog *P*. Decreasing the lipophilicity may be beneficial for potent MRSA inhibitory activity. Both compounds 5 and 6 had the structure of benzodioxole. Compound 5 with two hydroxyl moieties showed considerable anti-MRSA activity. The replacement of the hydroxyl groups with methoxyl groups (compound 6) eliminated the activity. The hydroxyl group or lower lipophilicity was required for benzodioxole to preserve the antimicrobial action. The increased hydroxyl group number and hydrophilicity were favorable for MRSA constraint as in the cases of compounds 7 and 8. The molecular size, hydrogen-bond number, and total polarity surface were not important to governing the anti-MRSA effect.

CoQ_0_ is one of the predominant pigments in *A. cinnamomea*. It belongs to the family of coenzyme Q consisting of a benzoquinone structure conjugated to an isoprenoid chain from C0 to C10. CoQ_0_ has the potential activity to suppress inflammation, cancer, and metabolic diseases (Wang et al., 2017). The inhibitory effect of CoQ_0_ on *S. aureus* development has also been reported in previous studies (Fan et al., 2019). CoQ_0_ showed the ability to suppress the growth of both MRSA and VISA. Since the values of MIC and MBC were approximate for MRSA and VISA treated with CoQ_0_, this compound can be classified as a bactericidal agent. The biofilm form of MRSA displayed differential gene expression and physiology compared to their planktonic form (Abouelhassan et al., 2018). The biofilm produced a barrier for antibiotic penetration, resulting in the further enhancement of drug resistance. CoQ_0_ was active in preventing biofilm MRSA growth. In the final stage of biofilm maturation, some bacteria were dispersed from the biofilm (Chung and Toh, 2014). Our results also showed that CoQ_0_ completely eradicated MRSA outside the biofilm. The amounts of proteins, RNA, and DNA of MRSA decreased after CoQ_0_ treatment. The results of live/dead fluorescence microscopy, biofilm visualization, SEM, and TEM excluded MRSA membrane damage by CoQ_0_. The genes related to transcription and translation, as well as the metabolic process involved in protein, RNA, and DNA synthesis, could be the anti-MRSA mechanisms of CoQ_0_. DNA polymerase and topoisomerases are the important determinants of MRSA viability and the persistence of infection (Lakhundi and Zhang, 2018). Our results suggested that the DNA polymerase inhibition plays a role in the ability of CoQ_0_ to impede MRSA growth.

CoQ_0_ also restrained the activity of topoisomerase I and gyrase. Both enzymes are categorized as belonging to the family of topoisomerases helping to regulate DNA topology. Topoisomerases are potential targets for many antibacterial agents (Tse-Dinh, 2016). CoQ_0_ may inhibit the topology of DNA during transcription, replication, and recombination. This is harmful to the survival of MRSA. The catalytic mechanism of DNA gyrase is involved in the double-strand DNA cleavage in an ATP-dependent manner (Chiriac et al., 2015). We found that some ATP-related proteins were increased by CoQ_0_ treatment. Under the harsh stress of the CoQ_0_ attack, the MRSA bacteria produced more energy to adapt to this adversity. The mode of action of this agent could be involved in the energy production. The proteomic assay also demonstrated a downregulation of ribosomes in CoQ_0_-treated pathogens. Ribosomes are the resistance proteins working against antibiotic drugs. A critical role of ribosomes is the maintenance of the substrate stability in the process of protein synthesis (Eyal et al., 2015). The inhibition of ribosomes by CoQ_0_ may be detrimental to the protein synthesis, resulting in the total-protein lessening as observed in our results. Some topoisomerases are quite different between prokaryotes and eukaryotes. Gyrase is even absent in higher eukaryotes (Chiriac et al., 2015). DNA topoisomerases are considered as an ideal target for anti-MRSA agents because of the minimal influence on mammalian cells. The multiple targeting to different enzymes by CoQ_0_ is advantageous to delaying the bacterial resistance because the possibility of simultaneous mutation on different targets is low.

It is worthwhile to develop the agents with combined antibacterial and anti-inflammatory actions to diminish bacteria-elicited inflammation (Gunasekaran et al., 2019). Keratinocytes are active in the immune reaction of AD by producing pro-inflammatory factors. CoQ_0_ has been reported as an anti-inflammatory agent in the cell models of endothelial cells and macrophages (Yang et al., 2015; Yang et al., 2016). The experimental results suggested that CoQ_0_ has an anti-inflammatory effect on stimulated keratinocytes through the inhibition of IL-6, CCL5, and CCL17. These cytokines and chemokines are all closely correlated to the pathogenesis and immune response of AD (Asahina and Maeda, 2017). CCL5 is an inflammatory chemokine produced in response to bacterial toxins (do Vale et al., 2016). CCL5 suppression is beneficial to limiting the inflammation caused by MRSA. The downregulation of TJ proteins in infected AD skin is a result of the inflammatory response (Bäsler and Brandner, 2017). TJ is involved in proliferation, differentiation, and adhesion in keratinocytes. The differentiation biomarkers filaggrin, involucrin, and integrin β1 are principal proteins in the construction of the skin-barrier property (Crawford and Dagnino, 2017). CoQ_0_ could ameliorate the deficiency of filaggrin and integrin β1 but not involucrin in 1649b-treated HaCaT. This indicates the capability of CoQ_0_ to protect the skin’s barrier function. This inference was verified by the TEWL and filaggrin expression in the *in vivo* mouse model.

A prerequisite for a topically applied agent showing therapeutic potential is effective permeation into the skin. Our data demonstrated the ability of CoQ_0_ to penetrate into the animal skins. The small size of CoQ_0_ (molecular weight = 182 Da) made facile permeation feasible. An optimized hydrophilic and lipophilic balance of the permeant structure is essential for achieving facile skin absorption. The higher lipophilicity causes greater delivery across the SC; however, this property may remain the permeant in the SC because of the retardation of further diffusion into viable skin (Lin et al., 2016). Yamaguchi et al. (2008) claimed the hindrance of viable skin transport for the permeant with log *P* > 2. CoQ_0_ with a log *P* of 0.51 would be suitable to diffuse across both the SC and viable skin. We found that OVA treatment greatly increased the skin penetration of CoQ_0_. Impairment of TJ led to an aberrant SC barrier associated with filaggrin inhibition and TEWL extension in the case of AD-like skin.

Immunization of the mice with OVA evokes cutaneous inflammation resembling AD (Kabashima, 2013). The presence of MRSA further increased the severity of the skin inflammation and barrier-function deficiency. The downregulation of TJ in AD is a trigger of increased *S. aureus* invasion (Ong and Leung, 2016). The antimicrobial peptides derived by keratinocytes are finite in AD skin (Hon et al., 2013). MRSA facilely invaded the AD lesions to produce significant colonization as detected in this study. The MRSA accumulation in the skin further damaged the epidermal barrier to allow allergen transport. AD lesions are characterized by a vicious cycle of disrupted barrier and cutaneous inflammation. The intervention to repair the barrier characteristics is useful for delaying AD progression (Udkoff et al., 2017). In the present study, topical CoQ_0_ protected the cutaneous barrier in AD-like skin. The upregulated CCL5 and CCL17 in AD not only caused the impaired integrity of the skin barrier but also induced immune-cell infiltration (Peng and Novak, 2015). Our results demonstrated that CoQ_0_ markedly diminished chemokine upregulation in keratinocytes to ameliorate the barrier function and the severity of AD inflammation. The chemokine suppression also impeded the infiltration of neutrophils to the dermis. The antibacterial activity of CoQ_0_ also restrained the MRSA load in AD-like skin. Our results illustrated that combined OVA and MRSA interventions significantly increased the expression of pro-inflammatory cytokines in mouse skin. Topically applied CoQ_0_ proved to limit the cytokines in the AD-like mouse model with MRSA invasion. This inhibition may have contributed to the amelioration of AD by decreasing immune-cell infiltration.

A concern pertaining to the application of CoQ_0_ is the safety of use. We found a cytotoxicity toward keratinocytes treated by CoQ_0_ at higher concentrations (≥7.8 µg/ml). It is reported that CoQ_0_ is toxic to mammalian cells through direct alkylation to DNA (Takahashi et al., 2018). Although CoQ_0_ showed some toxicity in the cell model, the *in vivo* skin-tolerance study manifested slight-to-mild skin irritation because the barrier function was disturbed in healthy mice. Whether CoQ_0_ exhibits a safe therapeutic index remains to be elucidated. A future strategy to resolve the irritation could be the design and optimization of the vehicles for CoQ_0_ to shield the possible toxicity. For AD management, combined corticosteroids and antibiotics have reportedly been successful (Fleischer, 2019). However, topical corticosteroids induce skin thinning to compromise skin damage and increase the sensitivity to allergens and *S. aureus*. The use of multiple drugs usually contributes to patient noncompliance. The combined anti-MRSA and anti-inflammatory activities of CoQ_0_ may be useful to resolving these drawbacks.

## Conclusions

The resulting compound from *A. cinnamomea* was tested for possible candidacy as an anti-AD agent capable of eradicating MRSA and suppressing skin inflammation. The results indicated that CoQ_0_ has a potential anti-MRSA effect against both the planktonic and biofilm forms. The antibacterial mechanism of CoQ_0_ could be the inhibition of DNA polymerase and topoisomerases. The proteomic analysis also exhibited a significant decrease of ribosomal proteins in the MRSA treated by CoQ_0_. A downregulation of cytokines and chemokines and the recovery of TJ-related proteins were observed in stimulated keratinocytes after CoQ_0_ treatment. Facile CoQ_0_ delivery into the skin led to the amelioration of AD-like lesions in an *in vivo* model. This compound also reduced the MRSA burden in the experimental AD skin. Taken together, our findings suggest the potential of topical CoQ_0_ as a new therapeutic option for the treatment of bacteria-infected AD.

## Data Availability Statement

The datasets for this study can be found in jPOSTrepo. The accession number is JPST000643.

## Ethics Statement

The animal study was reviewed and approved by Institutional Animal Care and Use Committee of Chang Gung University.

## Author Contributions

S-CY initiated the study and drafted the manuscript. J-YF, T-HH, and W-LC involved in the design of all experiments. W-LC, T-HH, P-WW, and Y-PC carried out the experiments. T-HL and J-YF analyzed data and wrote the manuscript. S-CY supervised the entire project. C-CC and Z-YC reviewed critically and approved the final manuscript. All authors read and approved the final manuscript.

## Conflict of Interest

The authors declare that the research was conducted in the absence of any commercial or financial relationships that could be construed as a potential conflict of interest.
