# Supplementary figures and images for: Coenzyme Q0 From Antrodia cinnamomea Exhibits Drug-Resistant Bacteria Eradication and Keratinocyte Inflammation Mitigation to Ameliorate Infected Atopic Dermatitis in Mouse
Source: Front Pharmacol. 2019 Dec 3;10:1445. doi: 10.3389/fphar.2019.01445 (PMC6901829; doi:10.3389/fphar.2019.01445)

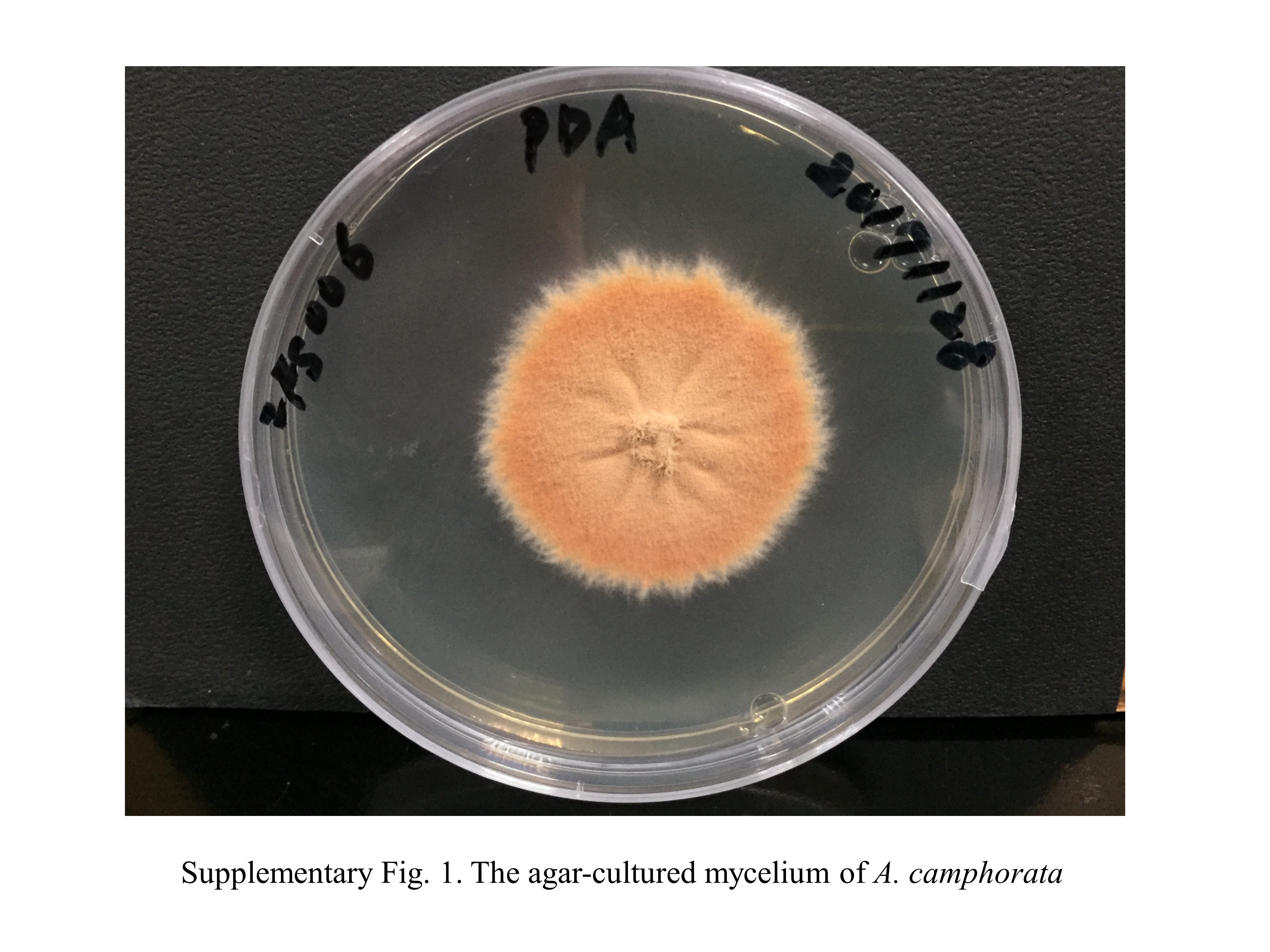

Supplement: Supplementary file 1 [file Image_1.tif]

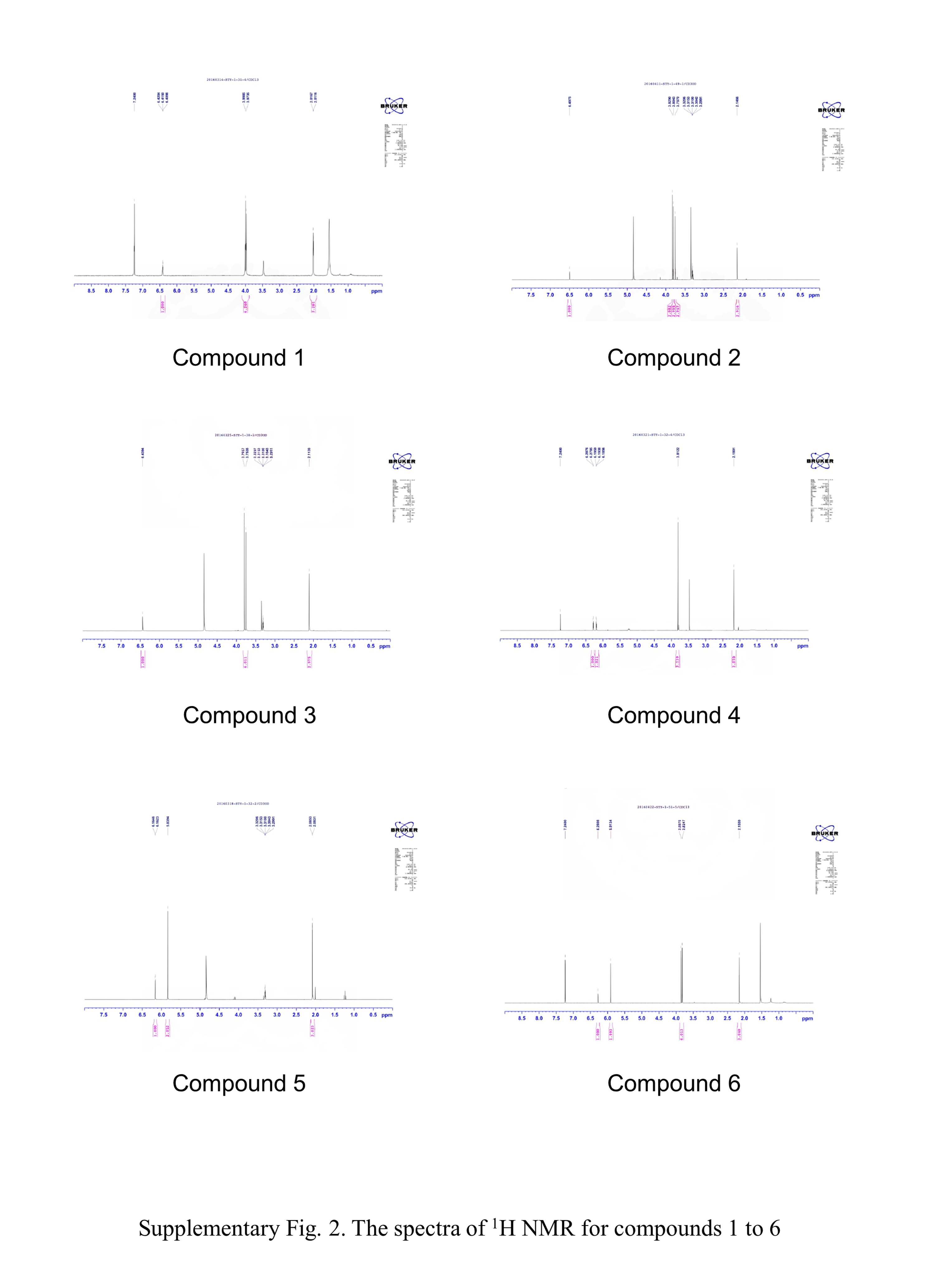

Supplement: Supplementary file 2 [file Image_2.tif]
