# Supplementary material for: Coenzyme Q0 From Antrodia cinnamomea Exhibits Drug-Resistant Bacteria Eradication and Keratinocyte Inflammation Mitigation to Ameliorate Infected Atopic Dermatitis in Mouse
Source: Front Pharmacol. 2019 Dec 3;10:1445. doi: 10.3389/fphar.2019.01445 (PMC6901829; doi:10.3389/fphar.2019.01445)
Supplement: Supplementary file 3 [file Table_1.docx]

Supplementary Table. 1 The Alog *P*, hydrogen bond number, and total polarity surface of benzenoids from *Antrodia cinnamomea* determined by molecular modeling

| Compound | Molecular weight (Da) | Alog *P* | Hydrogen-bond acceptor number | Hydrogen-bond donor number | Total polarity surface |
| --- | --- | --- | --- | --- | --- |
| Compound 1 | 184.19 | 1.799 | 4 | 2 | 58.92 |
| Compound 2 | 198.22 | 2.025 | 4 | 1 | 47.92 |
| Compound 3 (CoQ_0_) | 182.17 | 0.510 | 4 | 0 | 52.60 |
| Compound 4 | 154.16 | 1.815 | 3 | 2 | 49.69 |
| Compound 5 | 168.15 | 1.600 | 4 | 2 | 58.92 |
| Compound 6 | 196.20 | 2.051 | 4 | 0 | 36.92 |
